# Palmitate induces fat accumulation via repressing FoxO1-mediated ATGL-dependent lipolysis in HepG2 hepatocytes

**DOI:** 10.1371/journal.pone.0243938

**Published:** 2021-01-15

**Authors:** Naiqian Zhao, Huiwen Tan, Li Wang, Le Han, Yanli Cheng, Ying Feng, Ting Li, Xiaoling Liu

**Affiliations:** 1 Department of Gerontology, Second Hospital of Shanxi Medical University, Taiyuan, Shanxi, China; 2 Department of Endocrinology and Metabolism, West China Hospital of Sichuan University, Chengdu, Sichuan, China; East Tennessee State University, UNITED STATES

## Abstract

Obesity is closely associated with non-alcoholic fatty liver disease (NAFLD), and elevated serum palmitate is the link between obesity and excessive hepatic lipid accumulation. Forkhead box O-1 (FoxO1) is one of the FoxO family members of transcription factors and can stimulate adipose triglyceride lipase (ATGL) and suppress its inhibitor G0/G1 switch gene 2 (G0S2) expression in the liver. However, previous researches have also shown conflicting results regarding the role of FoxO1 in hepatic lipid accumulation. We therefore examined the role of FoxO1 as a downstream suppressor to palmitate-stimulated hepatic steatosis. Palmitate significantly promoted lipid accumulation but inhibited lipid decomposition in human HepG2 hepatoma cells. Palmitate also significantly reduced FoxO1, ATGL and its activator comparative gene identification-58 (CGI-58) expression but increased peroxisome proliferator-activated receptorγ (PPARγ) and its target gene G0S2 expression. FoxO1 overexpression significantly increased palmitate-inhibited ATGL and CGI-58 expression but reduced palmitate-stimulated PPARγ and its target gene G0S2 expression. FoxO1 overexpression also inhibited lipid accumulation and promoted lipolysis in palmitate-treated hepatocytes. Overall, these results indicate that FoxO1-mediated ATGL-dependent lipolysis may be an effective molecular mechanism in protecting hepatocytes from palmitate-induced fat accumulation.

## Introduction

During the past few decades, obesity has been sharply increasing in developed and developing countries, and is now a worldwide epidemic [1]. The global trend of obesity has led to a very high prevalence of obesity-associated non-alcoholic fatty liver disease (NAFLD) [2]. Hepatic lipid accumulation is the initial stage of NAFLD. Individuals with hepatic lipid accumulation may develop to nonalcoholic steatohepatitis, fibrosis, cirrhosis and possibly hepatocellular carcinoma progressing from simple steatosis [3]. Moreover, multiple evidences have indicated that NAFLD is associated with not only increased risk of developing type 2 diabetes mellitus (T2DM), but also with poor blood glucose control and an increased risk of cardiovascular disease/chronic kidney disease in patients with established T2DM [4]. Thus, the molecular mechanism promoting hepatic lipid accumulation in obesity is of biomedical and public health interest.

Obesity is connected with increased total concentrations of serum free fatty acids (FFAs) that have been released by subcutaneous adipose tissue with impaired lipid storage capacity. This creates an overall enhanced hepatic supply of circulating FFAs and subsequently leads to ectopic fat deposition in the liver [5,6]. The saturated fatty acid palmitate is one of the most abundant FFAs in both human and rodent plasma [7]. It can facilitate lipid accumulation in hepatocytes, characterized by a decreased rate of fat mobilization [8–11].

Fat mobilization is precisely regulated by a number of enzymes and factors. Adipose triglyceride lipase (ATGL) specifically catalyses the cleavage fracture of the first ester bond in triglycerides, which is the first and most critical rate-limiting step in intracellular triglyceride hydrolysis [12]. ATGL activity is mainly regulated by two regulatory protein, which are comparative gene identification-58 (CGI-58) and G0/G1 switching gene-2 (G0S2), respectively [13]. ATGL is activated by association with CGI-58, which introduces the targeting of ATGL to the surface of lipid droplets [14]. On the contrary, the protein product encoded by G0S2 is a specific inhibitor of ATGL-mediated lipolysis [15]. There is an direct interplay between G0S2 and the N-terminal patatin domain of ATGL, thus inhibiting ATGL triglyceride hydrolase activity and attenuating ATGL-mediated fat decomposition in adipose tissue and other tissues [13–15]. Specifically, recent studies showed that G0S2 is critical for triglyceride accumulation in the liver and is an important contributor to the development of liver steatosis [16,17]. Transactivation, gel shift and chromatin immunoprecipitation assessment revealed that the G0S2 promoter encompasses a potential peroxisome proliferator-activated receptor (PPAR)-response element (PPRE), and G0S2 is a direct PPARγ target gene [18]. When co-transfected in human HepG2 hepatoma cells, PPARγ directly binds to the PPRE sequence and markedly increases the reporter activity forced by the G0S2 promoter [18]. Likewise, the activation of PPARγ by rosiglitazone, a PPARγ agonist, remarkably up-regulates G0S2 protein expression in vitro [15].

Forkhead box O family proteins (FoxOs) of transcription factors are required for a large number of cellular processes, including antioxidant response, cell cycle control, glucose and lipid metabolism, apoptosis, and autophagy [19]. FoxOs contain four proteins, namely FoxO1 (also described as FKHR), FoxO3a (FKHRL1), FoxO4 (AFX1) and FoxO6, encoded by a distinct gene in mammalian cells respectively. Among them, FoxO1 is copiously expressed in the liver, pancreas, skeletal muscle and adipose tissue, typical tissues that affect energy homeostasis of the whole body [20]. During the past decade, FoxO1 has been identified as a direct transcriptional regulator of many genes that control many liver functions, including glucose heterogenesis and lipid metabolism [21]. FoxO1 plays an important role in regulating hepatic lipid metabolism. Adenoviral conveyance of constitutively active FoxO1 (CA-FoxO1) to primary mouse hepatocytes promotes depletion of hepatic triglyceride deposition that is associated with increased fatty acid oxidation [22]. Furthermore, CA-FoxO1 can simultaneously prompt ATGL and repress G0S2 expression in primary mouse hepatocytes [22]. FoxO1 also regulates the activity and expression of PPARγ, which is one of master adipogenic transcription factors. It interferes with PPARγ activity by competitively suppressing the formation of the PPARγ/retinoic X receptor functional complex in cultured adipocytes. On the other hand, FoxO1 directly binds to PPARγ promoter to repress its transcriptional activity, resulting in decreased PPARγ expression [23–25]. However, previous researches have also showed conflicting results with respect to the role of FoxO1 in hepatic lipid metabolism, and it is unclear how FoxO1 is implicated in palmitate-induced hepatic lipid accumulation.

Based on these previous findings, we put forward the undermentioned supposition: (1) palmitate suppresses FoxO1 expression and subsequently promotes PPARγ and G0S2 expression and suppresses ATGL expression, (2) the inhibition of ATGL-dependent lipolysis is conducive to palmitate-stimulated fat accumulation in the liver, and (3) FoxO1 overexpression reduces G0S2 and increases ATGL expression, accordingly having a preventive effect against palmitate-stimulated hepatic steatosis. Therefore, we investigated the functioning significance of FoxO1, PPARγ, G0S2, ATGL and CGI-58 in the human HepG2 hepatoma cell line treated with palmitate. HepG2 hepatocytes were selected because they are widely used as cell models for studying hepatic steatosis [26]. We examined alterations in the expression of these important regulatory proteins for ATGL activity and carried out gain of function analysis via vector-based protein overexpression. Our findings will facilitate our understanding with regard to the molecular mechanism by means of which palmitate promotes lipid accumulation in hepatocytes.

## Materials and methods

### Cell culture

HepG2 cells, a human hepatocellular carcinoma cell line (China Center for Type Culture Collection, Wuhan, China), were cultivated in Eagle’s medium modified by Dulbecco (DMEM; Invitrogen, Carlsbad, CA, US) supplemented with 100 μg/mL streptomycin, 100 U/mL penicillin, 1% L-glutamine and 10% foetal bovine serum (FBS; Invitrogen). The cells were incubated in cell culture containers at 37°C under a moisturized ambience with 5% CO_2_ and 95% air until use. The influence of palmitate was investigated by adding this reagent to cells covered in 6-well plates at 2 × 10^5^ cells/well.

### Palmitate solution preparation

As previously described [27], the treatment solution of palmitate (Sigma, St. Louis, MO, US) was made by combining the palmitate with bovine serum albumin (BSA; Sigma). In short, palmitate was fully melted in 200-proof ethanol for a concentration of 195 mM, which was added to a pre-warmed BSA solution (10% w/w, 37°C) to let palmitate tie to albumin and reach a final palmitate concentration of 3 mM. The ethanol concentration in the solution of palmitate did not go beyond 0.5% by volume. Palmitate was thoroughly dissolved by heating the mixture in a water bath at 37°C for an extra 10 min. The final molar ratio of BSA to palmitate was 1:2. The control solution was made using a stock of 10% w/w BSA with an equal volume of ethanol put in to match that included in the final palmitate stock. The final ethanol concentration in all experiments was less than 0.2% by volume.

### Quantitative real-time PCR

Total RNA was isolated from cultured HepG2 cells by using TRIzol^®^ reagent (Invitrogen). The integrity and quantity of total RNA were assessed using a Nanodrop ND1000 spectrophotometer (Thermo Scientific, Wilmington, DE, US). Total RNA (500 ng) was converted to cDNA using SuperScript II reverse transcriptase (Invitrogen) following the supplier’s recommendation. For each cDNA synthesis, reverse transcription conditions were 42°C for 15 min, 85°C for 5 s, and maintained at 4°C. The final products were kept at -20°C. The quantitative PCR (qPCR) of gene expression was performed by a StepOnePlus^TM^ Real-Time PCR System (Applied Biosystems, Inc., Foster City, CA, US), and the detection dye was SYBR Green. The primer sequences produced for PCR are shown in Table 1. β-Actin was designated as an endogenous control. Amplification procedure contained 45 circles of 94°C for 5 s and 60°C for 30 s. The estimated length of PCR products was examined on the 2% agarose gel electrophoresis and stained with ethidium bromide. In order to identify the specificity of the augmented PCR product, a melting curve analysis was directly connected to the PCR assay in each specimen. Relative gene expression was analyzed by ΔΔC(t) method and emerges as the fold differences between each gene of interest and β-actin in the same sample.

**Table 1:** Primer pairs used in the qPCR analysis.

Primer name	Product size (bp)	Sequence (5′–3′)
FoxO1 (forward)	226	AATCCAGAGGGTGGCAAGAGCG
FoxO1 (reverse)		GGCGAAATGTACTCCAGTTATCAA
ATGL (forward)	163	GGACCTGCTGACCACCCTCT
ATGL (reverse)		CGTCTGCTCCTTCATCCACC
CGI-58 (forward)	162	TGGATTCTTGGCTGCTGCTTAC
CGI-58 (reverse)		TTAAAGGGAGTCAATGCTGCTC
PPARγ (forward)	169	ACCACTCCCACTCCTTTG
PPARγ (reverse)		GCAGGCTCCACTTTGATT
G0S2 (forward)	160	CCTCTTCGGCGTGGTGCT
G0S2 (reverse)		CTGCTGCTTGCCTTTCTCC
β-Actin (forward)	186	TGGCACCCAGCACAATGAA
β-Actin (reverse)		CTAAGTCATAGTCCGCCTAGAA

### Western blotting

Protein lysates were prepared from the cultured HepG2 cells employing ice-cold RIPA lysis buffer including protease inhibitor cocktail (Roche Diagnostics, Mannheim, Germany). Protein concentration was quantified by a Bio-Rad DC protein assay kit (Bio-Rad, Hercules, CA, US). In short, after heating to cause protein denaturation, total protein (40 μg) was loaded per lane and resolved by sodium dodecyl sulphate polyacrylamide gel electrophoresis (SDS-PAGE) (10% w/v) at room temperature for 2 h. Subsequently, proteins were transferred overnight to polyvinylidene difluoride (PVDF) membranes (Atto Corporation, Tokyo, Japan). Membranes were blocked using 5% non-fat milk powder in TBS with 0.05% Tween 20 for 2 h and detected using primary antibodies overnight, followed by incubation with a horseradish peroxidase secondary antibody for 1 h. The primary antibodies used were: anti-FoxO1 (1:600 dilution) (Santa Cruz Biotechnology, Santa Cruz, CA, US); anti-PPARγ (1:1000 dilution) (Cell Signalling, NEB, Vienna, Austria); anti-G0S2 (1:100 dilution) (Sigma); anti-CGI-58(1:1000 dilution) (Santa Cruz); anti-ATGL (1:1000 dilution) (Cell Signalling). An anti-β-actin antibody (1:2000 dilution) (Sigma) emerged as the internal control. Goat anti-rabbit IgG-horseradish peroxidase conjugate (1:2000 dilution) (Bio-Rad Ltd.) was applied as a secondary antibody. Membranes were visualized on a enhanced chemiluminescence detection system (Amersham Pharmacia Biotech, Piscataway, NJ, US) following the manufacturer’s protocol. The target protein bands were selected and determined to achieve signal intensities with ImageJ software (NIH, Bethesda, MD, US). The signal intensities of target protein bands were transformed and normalized to that of β-actin, which are described as the integral optical density (IOD) ratio.

### Plasmid-mediated overexpression

The FoxO1 overexpression plasmid (pReceiver-M98-FoxO1) and an empty plasmid (pReceiver-M98-empty) were custom-made synthesized by GeneCopoeia Inc. (Guangzhou, China). The pReceiver-M98-empty plasmid was used as a transfection reference. According to the manufacturer’s instructions, transient and stable plasmid transfection experiments were carried out by using cationic liposome Lipofectamine 2000 (Invitrogen) The cells were paved on 6-well culture plates at 1 × 10^5^ cells/well and incubated for an overnight stay. The cells were rinsed twice using fresh DMEM, and then the wells were packed with fresh DMEM (2.0 ml/well). pReceiver-M98-FoxO1 plasmid (2.5 μg), pReceiver-M98-empty plasmid (2.5 μg) and Lipofectamine 2000 reagent (5 μL) were scattered in 250 μL Opti-MEM^®^ Medium (Invitrogen) respectively and cultured separately at room temperature for 10 min. Plasmid dilution and Lipofectamine^®^ 2000 reagent were blended softly in equal volumes, and the mixtures were cultured at room temperature for another 10 min to develop plasmid-lipid complexes. Subsequently, the complexes were put into each well. The HepG2 cells were incubated at 37°C for 24 h, and then the medium was displaced with fresh DMEM including 10% FBS (2.0 mL/well). The cells were cultured for another 24 h until use. Total protein was collected and Western blot analysis was carried out to determine the transfection efficiency.

### Oil red O staining

HepG2 cells were planted on 6-well plates. After the treatment incubation, cells were fixed with 10% formaldehyde at room temperature for 30min. Subsequently, the cells were immersed by 60% isopropyl alcohol for 3 min, and dyed with 2 mg/mL oil red O (Sigma) staining reagent for 60 min. After coloring, the cells were flushed with ddH_2_O for three times to clear away any free dye. The cell nuclei were briefly counterstained in aqueous haematoxylin for 3 min. The pictures were captured by using an Axiovert 40 CFL microscope system (Olympus, Tokyo, Japan). After the microscopic image formation, intracellular triglycerides were quantified by detecting the amount of oil red O. The solution of isopropanol extraction (200 μL) was put into each well, and the liquid mixtures were softly shaken at room temperature for 10 min to liberate the oil red O dye. The extracted dye was removed by soft pipetting and its optical density was obtained at 500 nm using a microplate reader system (VersaMax, Molecular Devices, Sunnyvale, CA, US).

### Intracellular triglyceride assay

HepG2 cells were seeded into 6-well plates. After treatment, cells were collected, lysed with the lysis buffer. Triglycerides were measured by Triglyceride Assay Kit (Biovision, Inc., Milpitas, CA, US) according to the manufacturer's instructions. Total cellular protein content was quantified following the manufacturer's instructions of Pierce 660 nm Protein Assay Reagent (Thermo Fischer Scientific, Rockford, IL, US). Intracellular triglyceride concentration was normalized to total protein concentration and is expressed in μmol/g protein.

### Lipolysis measurement

Lipolysis was evaluated by measuring the concentration of glycerol and FFAs in the cell culture supernatant. Aliquots of culture media were collected and assayed for glycerol and FFAs using a Glycerol or Free Fatty Acid Quantification Kit (Biovision). Glycerol and FFAs were quantified by determining the optical density at 570 nm on a spectrophotometer (VersaMax) following the manufacturer’s instructions.

### Statistical analysis

All the observational data were expressed as the means *±* standard deviations (SDs). Statistical analysis was performed using one-way analysis of variance, followed by Student's *t*-test with SPSS 18.0 analysis software (SPSS, Chicago, IL, US). *P*<0.05 was considered to be statistically significant.

## Results

### Palmitate promoted triglyceride accumulation and inhibited lipolytic activity in HepG2 hepatocytes

**Fig 1:**
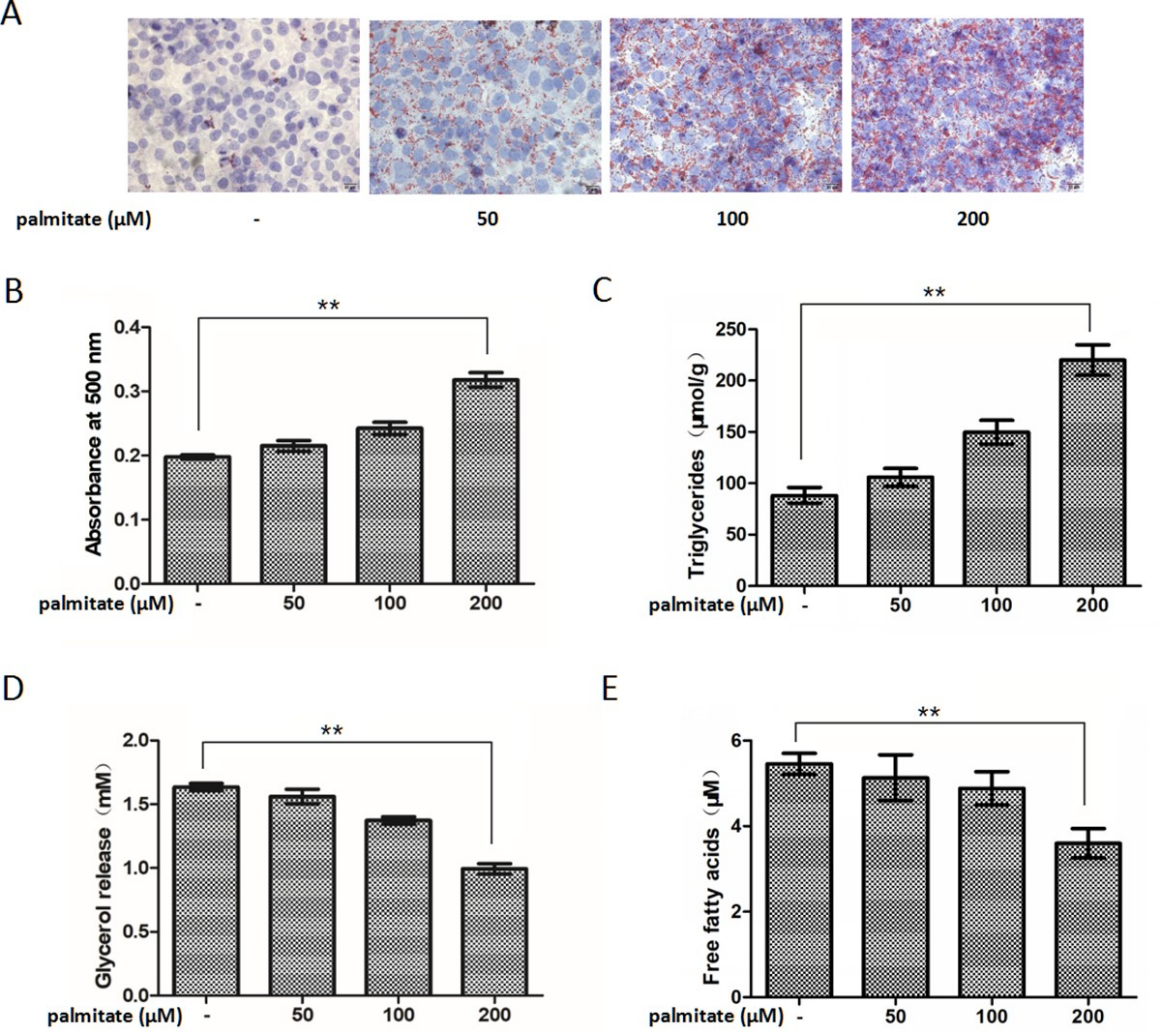
Palmitate promoted lipid accumulation and inhibited lipolytic activity in a concentration-dependent manner in HepG2 hepatocytes. Intracellular lipid accumulation was developed (A) by oil red O staining and (B) assessed by the optical density value of the collected oil red O dye. Original magnifying, 400×. Scale bars = 20 μm. (C) Intracellular lipid accumulation was measured by intracellular triglyceride assay. Lipolytic activity was determined by (D) the glycerol and (E) FFAs content in the cell media. Each bar represents the means ± SDs (n = 3). *P < 0.05, **P < 0.01.

HepG2 hepatocytes were exposed to increasing palmitate concentrations (0, 50, 100 or 200 μM) and harvested at 24 h, and the resulting triglyceride accumulation was tested by oil red O staining and intracellular triglyceride assay. Palmitate induced a concentration-dependent increase in triglyceride accumulation in HepG2 hepatocytes (Fig 1A–1C). The exposure of HepG2 hepatocytes to palmitate also caused a concentration-dependent decrease in lipolytic activity, as demonstrated by the glycerol and FFAs amount released into the cell media (Fig 1D and 1E). Moreover, palmitate with a dose of 200 μM, which stands for a high physiological level for the circulating palmitate concentrations of obese subjects [28], provided a remarkable increase in triglyceride accumulation and a significant decrease in lipolytic activity.

### Palmitate reduced FoxO1, ATGL and CGI-58 expression but increased PPARγ and G0S2 expression in HepG2 hepatocytes

**Fig 2:**
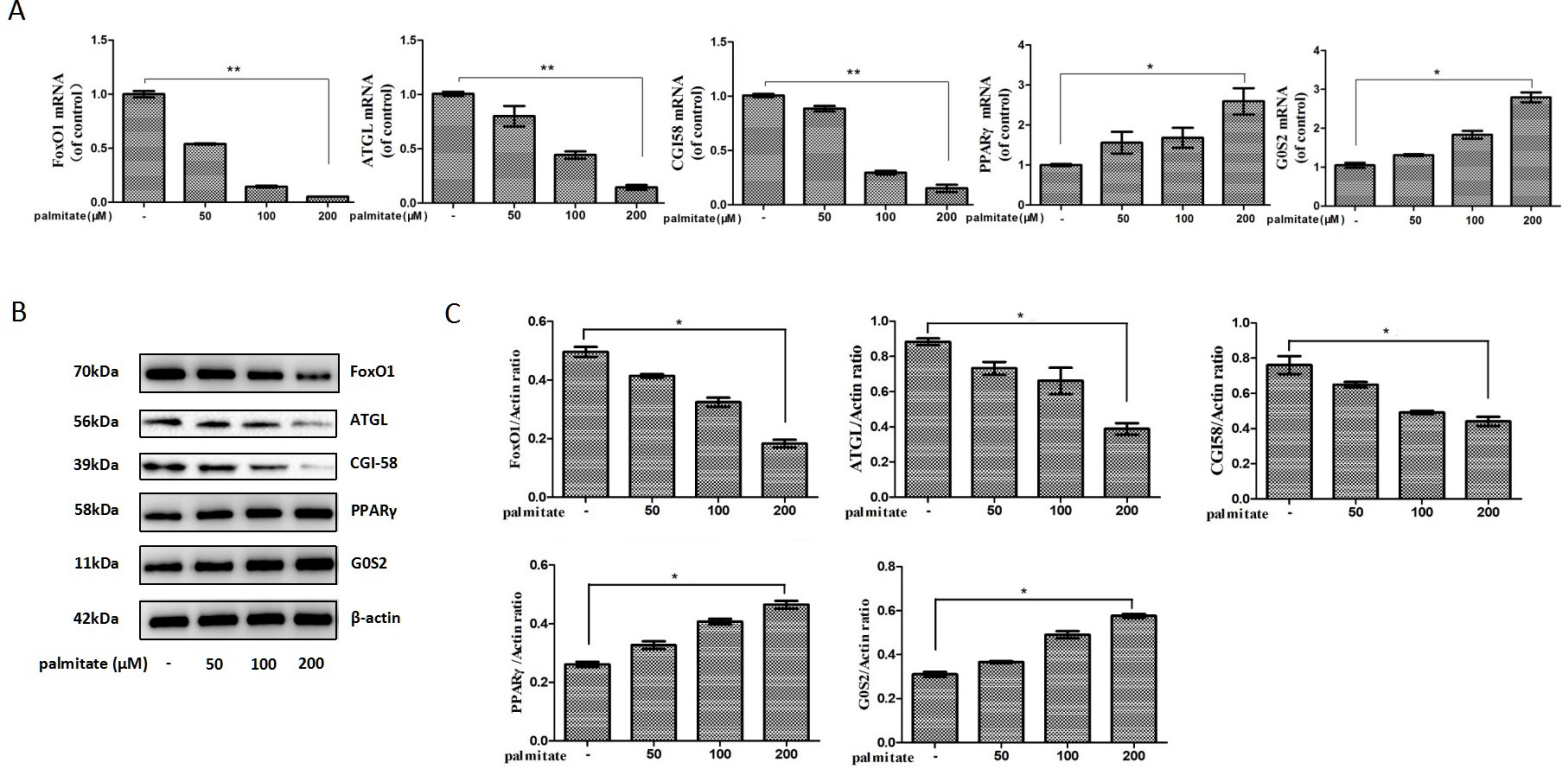
Palmitate reduced FoxO1, ATGL and CGI-58 and increased PPARγ and its target gene G0S2 expression in HepG2 hepatocytes in a concentration-dependent manner. (A) Palmitate reduced FoxO1, ATGL and CGI-58 but increased PPARγ and G0S2 mRNA expression. The mRNA levels were determined by a quantitative PCR analysis and were normalized to β-actin. (B) Palmitate reduced FoxO1, ATGL and CGI-58 and increased PPARγ and G0S2 protein expression. The protein expression levels were determined by a Western blot analysis. (C) The bands were quantified and normalized relative to β-actin. Each bar represents the means ± SDs (n = 3). *P < 0.05, **P < 0.01.

To investigate the underlying molecular mechanism of palmitate-stimulated hepatic triglyceride accumulation, the effects of palmitate on the expression of FoxO1, ATGL, CGI-58, PPARγ, and G0S2 were evaluated in HepG2 hepatocytes. We exposed HepG2 hepatocytes to increasing palmitate concentrations (0, 50, 100 or 200 μM) for 24 h. Palmitate caused a concentration-dependent decrease in FoxO1, ATGL and CGI-58 mRNA expression but a concentration-dependent increase in PPARγ and G0S2 mRNA expression (Fig 2A). Exposure of HepG2 hepatocytes to increasing palmitate concentrations for 24 h produced a concentration-dependent decrease in FoxO1, ATGL and CGI-58 protein expression but a concentration-dependent increase in PPARγ and G0S2 protein expression (Fig 2B and 2C).

### FoxO1 overexpression increased ATGL and CGI-58 expression but reduced PPARγ and G0S2 expression in palmitate-stimulated HepG2 hepatocytes

**Fig 3:**
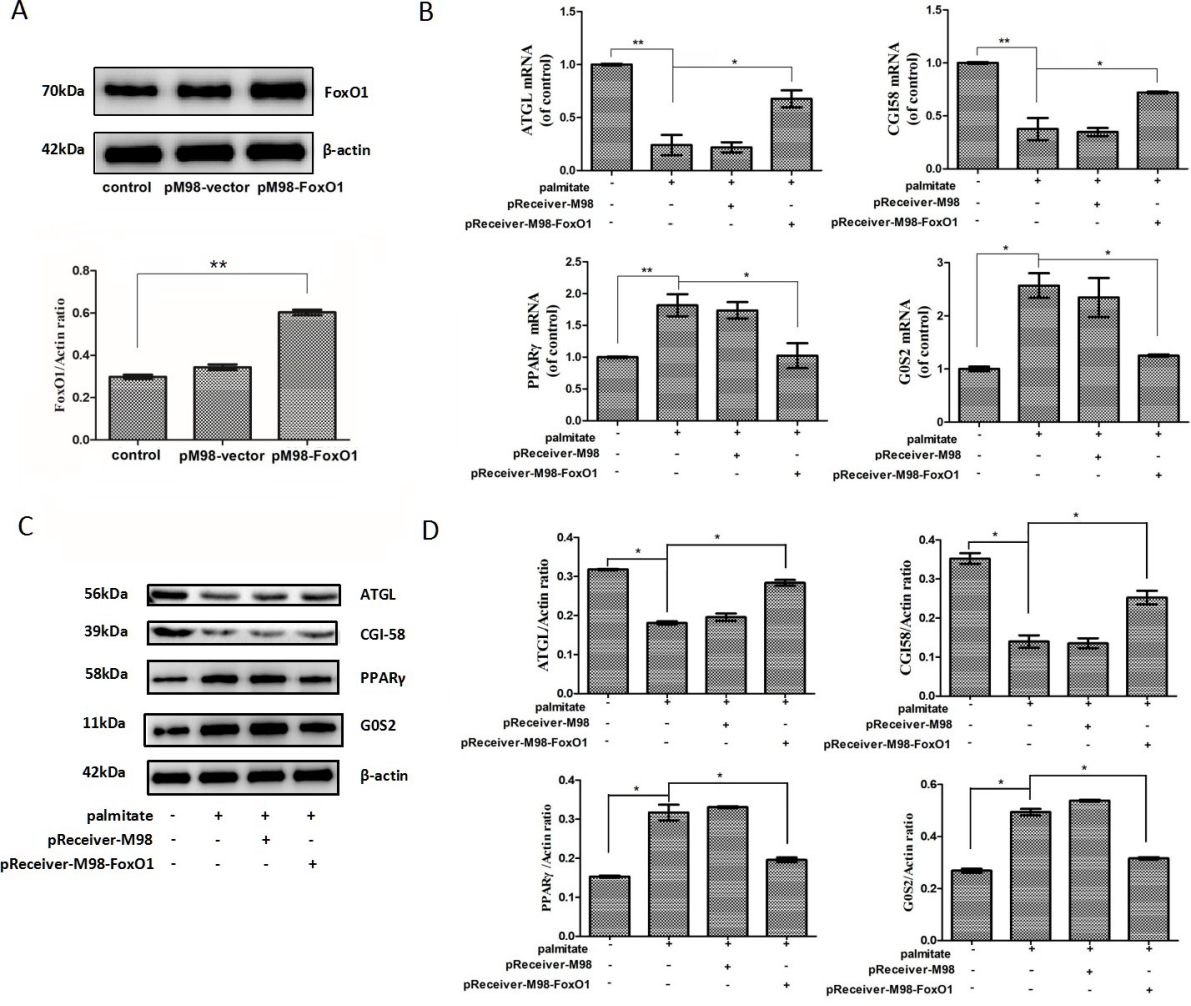
FoxO1 overexpression increased ATGL and CGI-58 but reduced PPARγ and G0S2 expression in palmitate-treated HepG2 hepatocytes. (A) HepG2 hepatocytes were transfected with pReceiver-M98-empty or pReceiver-M98-FoxO1, and FoxO1 expression was determined through a Western blotting analysis. (B) HepG2 hepatocytes were transfected with pReceiver-M98-FoxO1 to stably over-express FoxO1 and then exposed to 200 μM palmitate for 24 h. FoxO1 overexpression increased ATGL and CGI-58 but reduced PPARγ and G0S2 mRNA expression. The mRNA levels were determined by a quantitative PCR analysis and were normalized to β-actin. (C) FoxO1 overexpression increased ATGL and CGI-58 but reduced PPARγ and G0S2 protein expression. The protein expression levels were determined by a Western blot analysis. (D) The bands were quantified and normalized relative to β-actin. Each bar represents the means ± SDs (n = 3). *P < 0.05, **P < 0.01.

HepG2 hepatocytes were transfected with pReceiver-M98-FoxO1 to stably over-express FoxO1 and then exposed to 200 μM palmitate for 24 h. and then exposed to 200 μM palmitate for 24 h. FoxO1 protein expression was efficiently increased in the pReceiver-M98-FoxO1-transfected HepG2 cells (Fig 3A). FoxO1 overexpression remarkably increased ATGL and CGI-58 mRNA expression but reduced PPARγ and G0S2 mRNA expression (Fig 3B). Meanwhile, FoxO1 overexpression remarkably increased ATGL and CGI-58 protein expression but decreased PPARγ and G0S2 protein expression (Fig 3C and 3D).

### FoxO1 overexpression inhibited triglyceride accumulation and promoted lipolytic activity in palmitate-stimulated HepG2 hepatocytes

**Fig 4:**
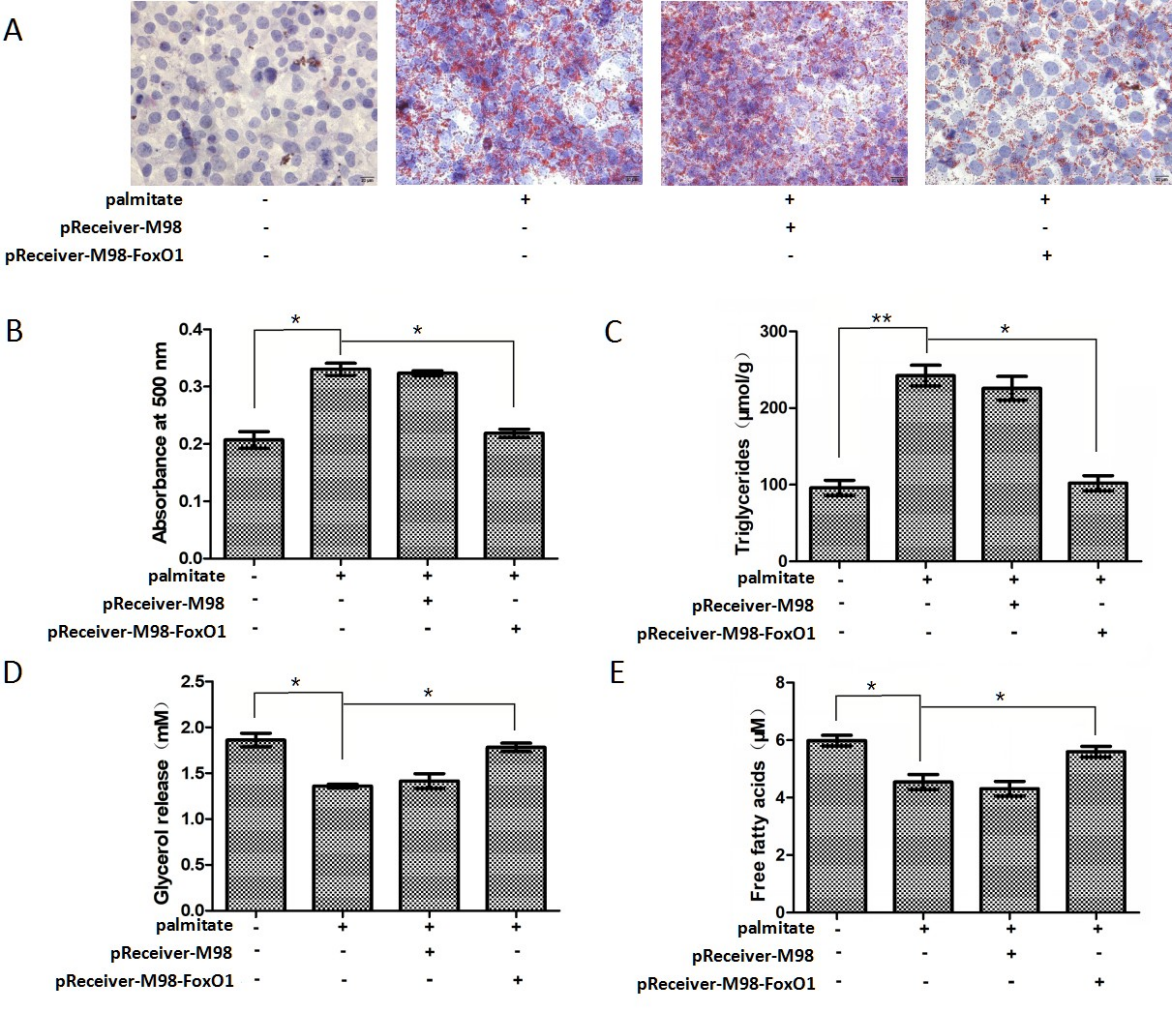
FoxO1 overexpression inhibited lipid accumulation and promoted lipolytic activity in palmitate-stimulated HepG2 hepatocytes. Intracellular lipid accumulation was developed (A) by oil red O staining and (B) assessed by the optical density value of the collected oil red O dye. Original magnifying, 400×. Scale bars = 20 μm. (C) Intracellular lipid accumulation was measured by intracellular triglyceride assay. Lipolytic activity was determined by (D) the glycerol and (E) FFAs content in the cell media. Each bar represents the means ± SDs (n = 3). *P < 0.05, **P < 0.01.

Since FoxO1 has been involved in triglyceride accumulation in hepatocytes [22], we focused on the effects of FoxO1 on triglyceride accumulation and lipolytic activity in palmitate-treated HepG2 hepatocytes as well. HepG2 hepatocytes were transfected with pReceiver-M98-FoxO1 and then exposed to 200 μM palmitate for 24 h. FoxO1 overexpression significantly inhibited palmitate-induced triglyceride accumulation (Fig 4A–4C) but promoted palmitate-inhibited lipolytic activity in HepG2 hepatocytes (Fig 4D and 4E).

## Discussion

Elevated plasma palmitate levels play an etiological part in the development of NAFLD and are presumed to make up a crucial connection between obesity and the risk of NAFLD [29]. Palmitate treatment has been previously demonstrated to lead to pronounced fat accumulation in hepatocytes [10,30]. In the present study, we also verified that palmitate promotes intracellular triglyceride accumulation and inhibits lipolytic activity in HepG2 hepatocytes in a concentration-dependent manner. However, the molecular trigger mechanisms through which palmitate is connected to triglyceride accumulation have not been thoroughly clarified.

The liver is the paramount organ for triglyceride metabolism [31]. The crucial role of FoxO1 in hepatic lipid metabolism is well set up by using genetically modified mice [32]. However, studies utilizing a variety of constitutive active mutations of FoxO1 protein seemed to imply both positive and negative results of FoxO1 on lipid formation and accumulation. One study showed that adenoviral conveyance of constitutively hyperactivated nuclear FoxO1 to mouse liver contributes to hepatic triglyceride accumulation [33]. The lipid accumulation is associated with reduced fatty acid oxidation and can advance to steatosis [33]. Conversely, another study indicated that the expression of CA-FoxO1 in primary hepatocytes from wild type mice results in decreased hepatic triglyceride accumulation [22]. Moreover, the expression of CA-FoxO1 stimulates ATGL and suppresses G0S2 expression, while in liver-specific FoxO knockout mice, hepatic ATGL expression is reduced and G0S2 expression is increased, demonstrating that FoxO1 regulates ATGL and G0S2 expression in a cell self-governing pattern [22]. Therefore, the exact effect of FoxO1 on hepatic lipid metabolism and its underlying molecular mechanism need to be unambiguously elucidated.

PPARγ is one of essential adipogenic transcription factors and plays a crucial role in adipogenesis [34]. Several studies have shown that FoxO1 causes an interference with PPARγ expression in cultured adipocytes [23–25]. FoxO1 binds to PPARγ promoter and inhibits its transcriptional activity, resulting in decreased PPARγ expression. what is more, FoxO1 haploinsufficiency also causes increased PPARγ gene expression in adipose tissue in vivo, accompanied by increased expression of PPARγ target genes recognized to affect metabolism [35]. In the present study, we found that palmitate reduces FoxO1 expression and increases PPARγ and its target gene G0S2 expression in HepG2 hepatocytes in a concentration-dependent manner. FoxO1 overexpression significantly reduces PPARγ and G0S2 expression in palmitate-stimulated HepG2 hepatocytes. Furthermore, FoxO1 overexpression significantly decreases triglyceride accumulation and increases lipolytic activity in palmitate-stimulated HepG2 hepatocytes.

Recent evidence showed that fatty acids come from adipose tissue induce hepatic G0S2 expression in the fasting state and lead to enhanced triglyceride deposition in the liver [36]. Fasting G0S2 protein expression displays an increase in liver but a decrease in adipose tissue [16]. G0S2 knockout causes a drastic decrease in hepatic triglyceride amount, whereas G0S2 overexpression prompts triglyceride accumulation and contributes to fatty liver production [16,17]. These findings, together with the above-mentioned study showing that FoxO1 suppresses G0S2 expression [22], revealed that palmitate promotes hepatic fat accumulation by disrupting the negative effect of FoxO1 on PPARγ-induced G0S2 expression.

ATGL is the major enzyme in charge of the first step in triglyceride mobilization. Previous evidence revealed that the ATGL promotor region has two FoxO1-binding sites, and FoxO1 can directly target the promoter of ATGL and promote ATGL expression in adipose tissue [37]. In the present study, we found that palmitate reduces FoxO1 and ATGL expression in HepG2 cells in a concentration-dependent manner, and FoxO1 overexpression significantly induces ATGL expression in palmitate-stimulated HepG2 hepatocytes, which is in agreement with the above-mentioned study showing that FoxO1 stimulates ATGL expression [22]. Therefore, our results indicated that FoxO1 overexpression plays a positive role in mediating and regulating effects of palmitate on ATGL expression in the liver.

CGI-58 is a potent activator of triglyceride hydrolysis. It can promote triglyceride hydrolysis by activating ATGL. One previous study showed that saturated non-esterified fatty acid suppresses CGI-58 expression in macrophages [38]. In the present study, we also showed that palmitate reduces FoxO1 and CGI-58 expression in HepG2 hepatocytes in a concentration-dependent manner. Especially, FoxO1 overexpression significantly increases CGI-58 expression in palmitate-stimulated HepG2 hepatocytes. Furthermore, using the web tool ECR Browser (https://ecrbrowser.dcode.org), we found that CGI-58 promotor region has a potential FoxO1 binding site, indicating that FoxO1 might also regulate and mediate effects of palmitate on CGI-58 expression in the liver. Further chromatin immunoprecipitation assay and luciferase reporter assay are needed to expound this problem thoroughly.

## Conclusions

**Fig 5:**
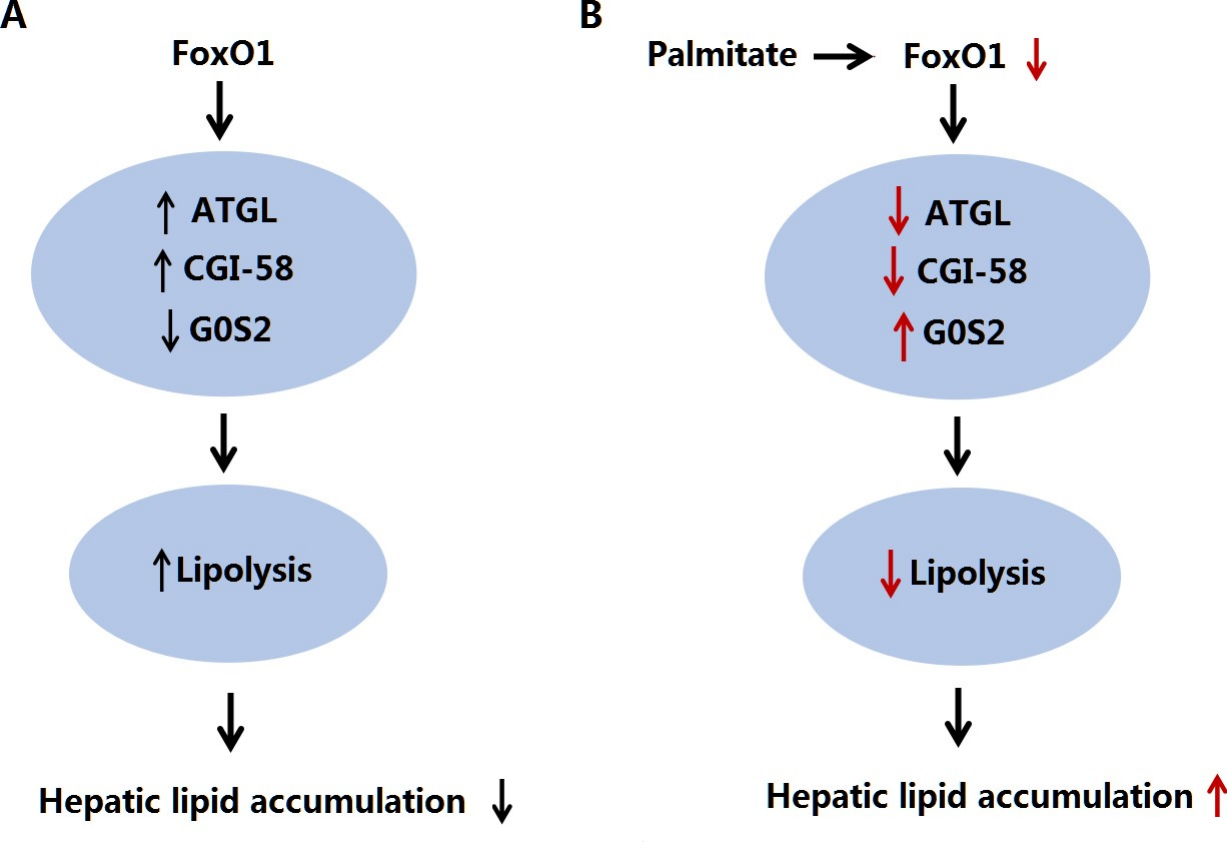
Proposed scheme for the effect and mechanism of FoxO1 in hepatic lipid metabolism. (A) FoxO1 stimulates ATGL and CGI-58 and suppresses G0S2 expression, and subsequently promotes intrahepatic lipolysis in an ATGL-dependent fashion. (B) Inhibition of FoxO1 expression by palmitate contributes to changes in hepatic triglyceride accumulation at least in part due to the inhibition of ATGL-dependent lipolysis.

In summary, the present study showed that FoxO1 simultaneously regulates ATGL, CGI-58 and G0S2 expression in HepG2 hepatocytes and revealed an important role for ATGL-dependent lipolysis in mediating the protective effect of FoxO1 on palmitate-stimulated excessive triglyceride accumulation (Fig 5). Induction of hepatic FoxO1 expression may provide an effective strategy for raising hepatic triglyceride catabolism, thereby probably preventing hepatic triglyceride accumulation and obesity-associated NAFLD.

## Supporting information

S1 Raw images (PDF)
