# Supplementary material for: Palmitate induces fat accumulation via repressing FoxO1-mediated ATGL-dependent lipolysis in HepG2 hepatocytes
Source: PLoS One. 2021 Jan 15;16(1):e0243938. doi: 10.1371/journal.pone.0243938 (PMC7810308; doi:10.1371/journal.pone.0243938)
Supplement: S1 Raw images — (PDF) [file pone.0243938.s001.pdf]

Figure 1

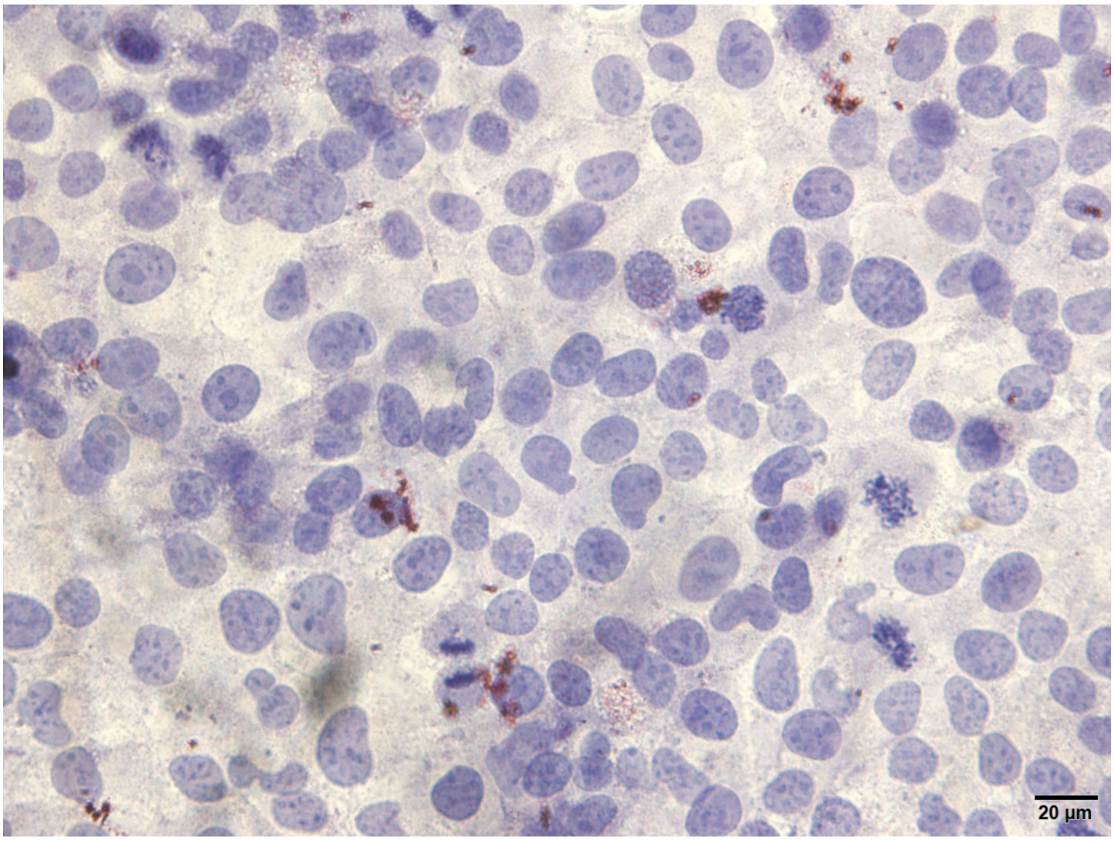

control

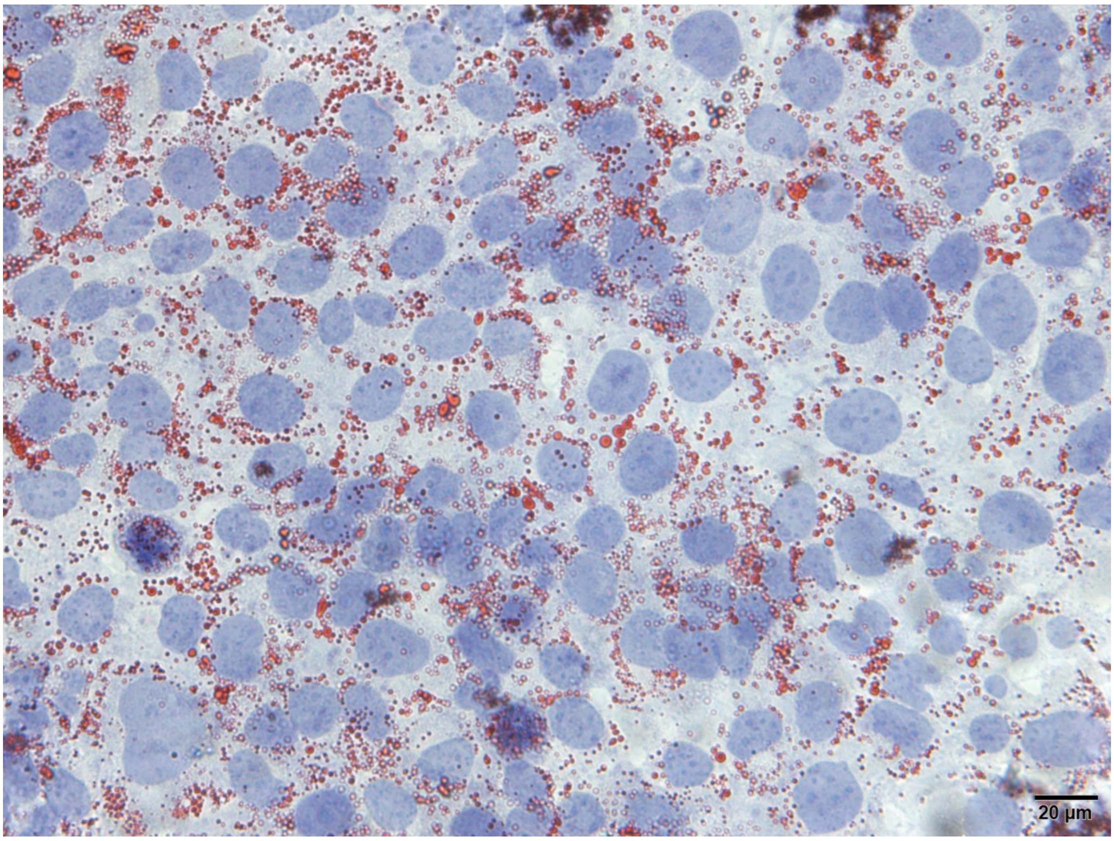

50µM palmitate

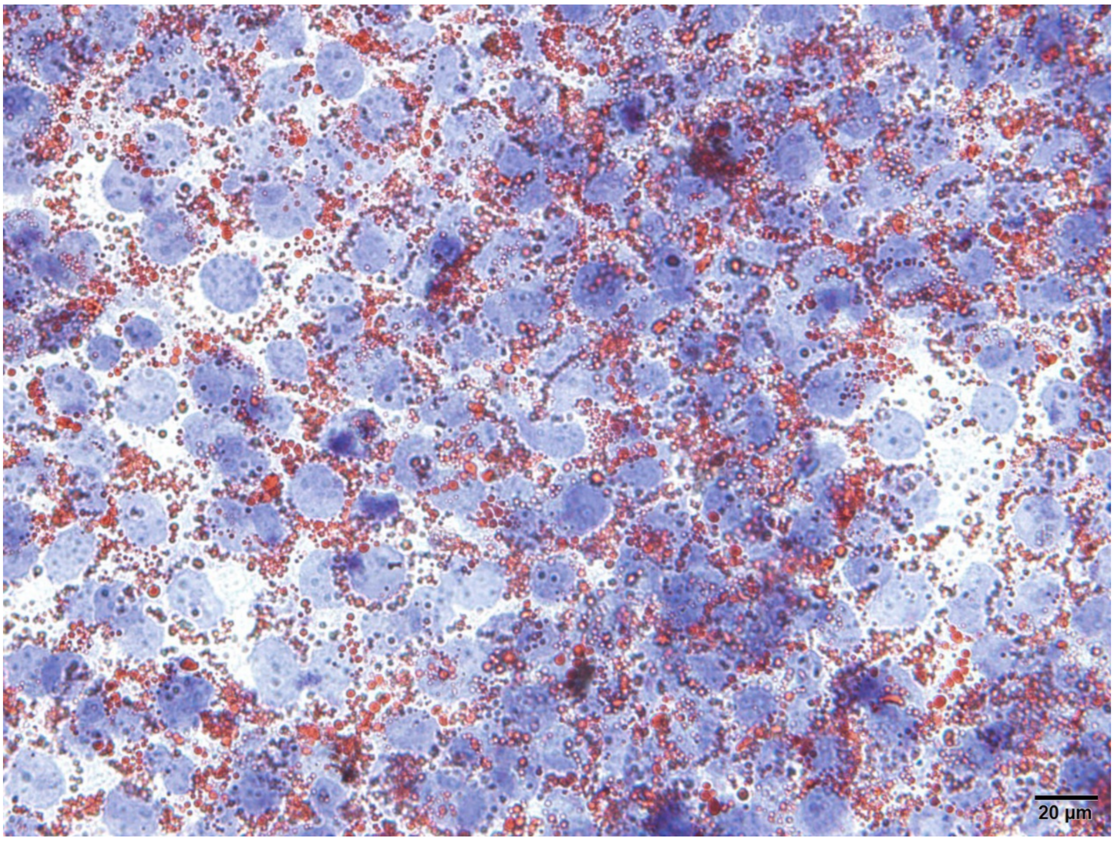

100µM palmitate

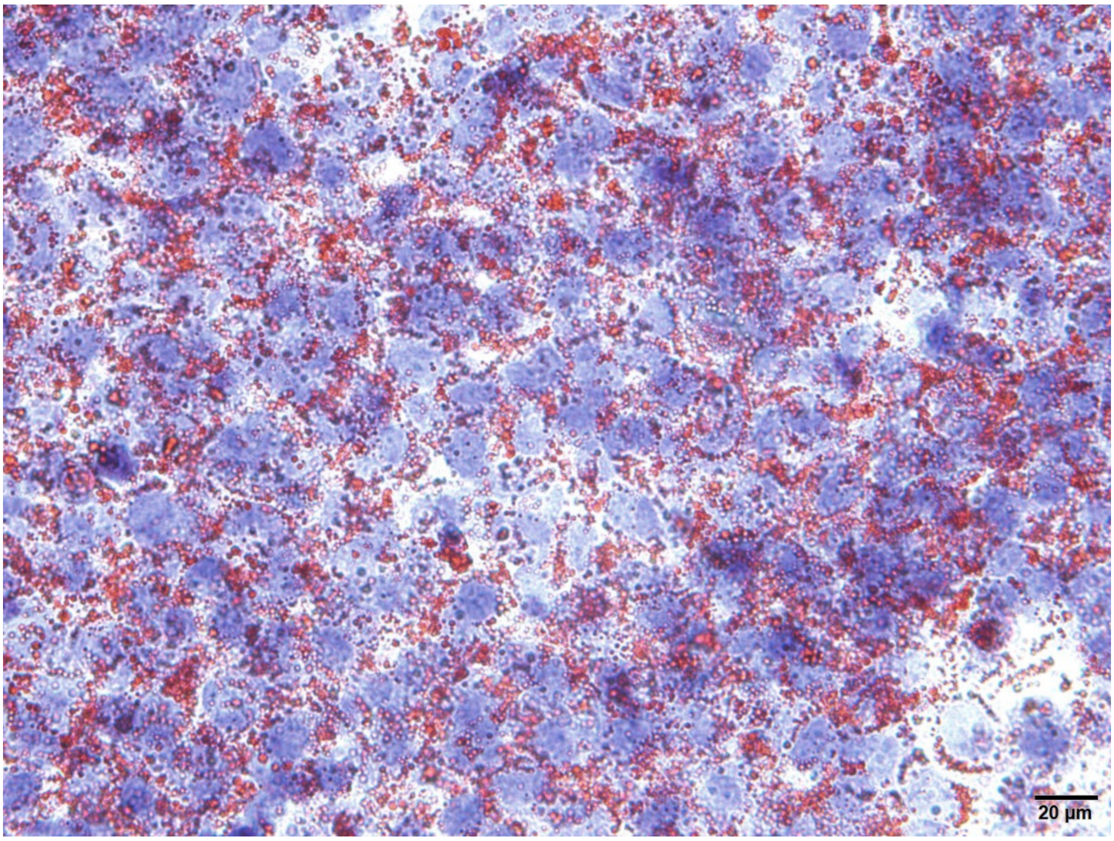

200µM palmitate

Figure 2

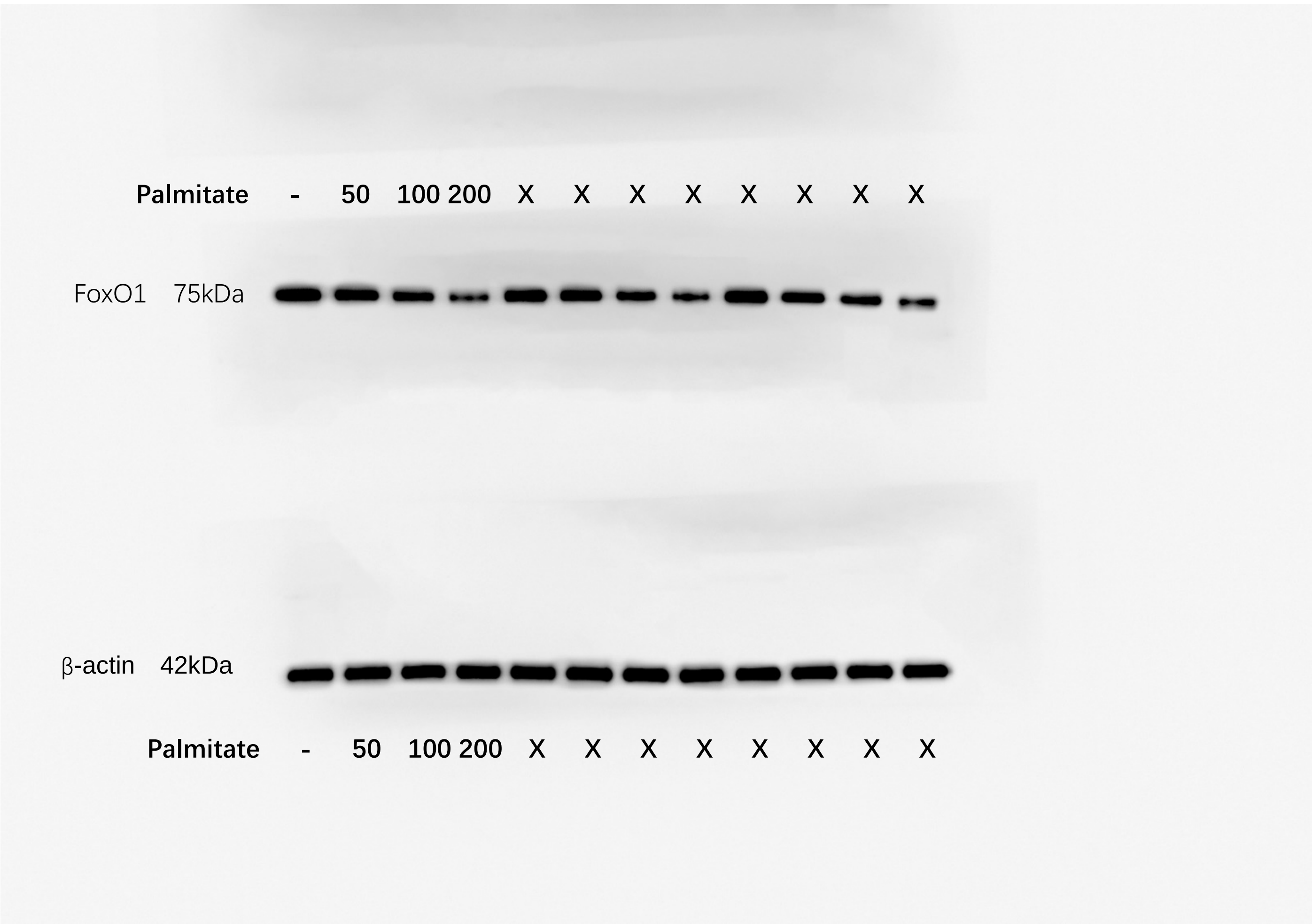
Palmitate - 50 100 200 X X X X X X X X
FoxO1 75kDa
β-actin 42kDa
Palmitate - 50 100 200 X X X X X X X X

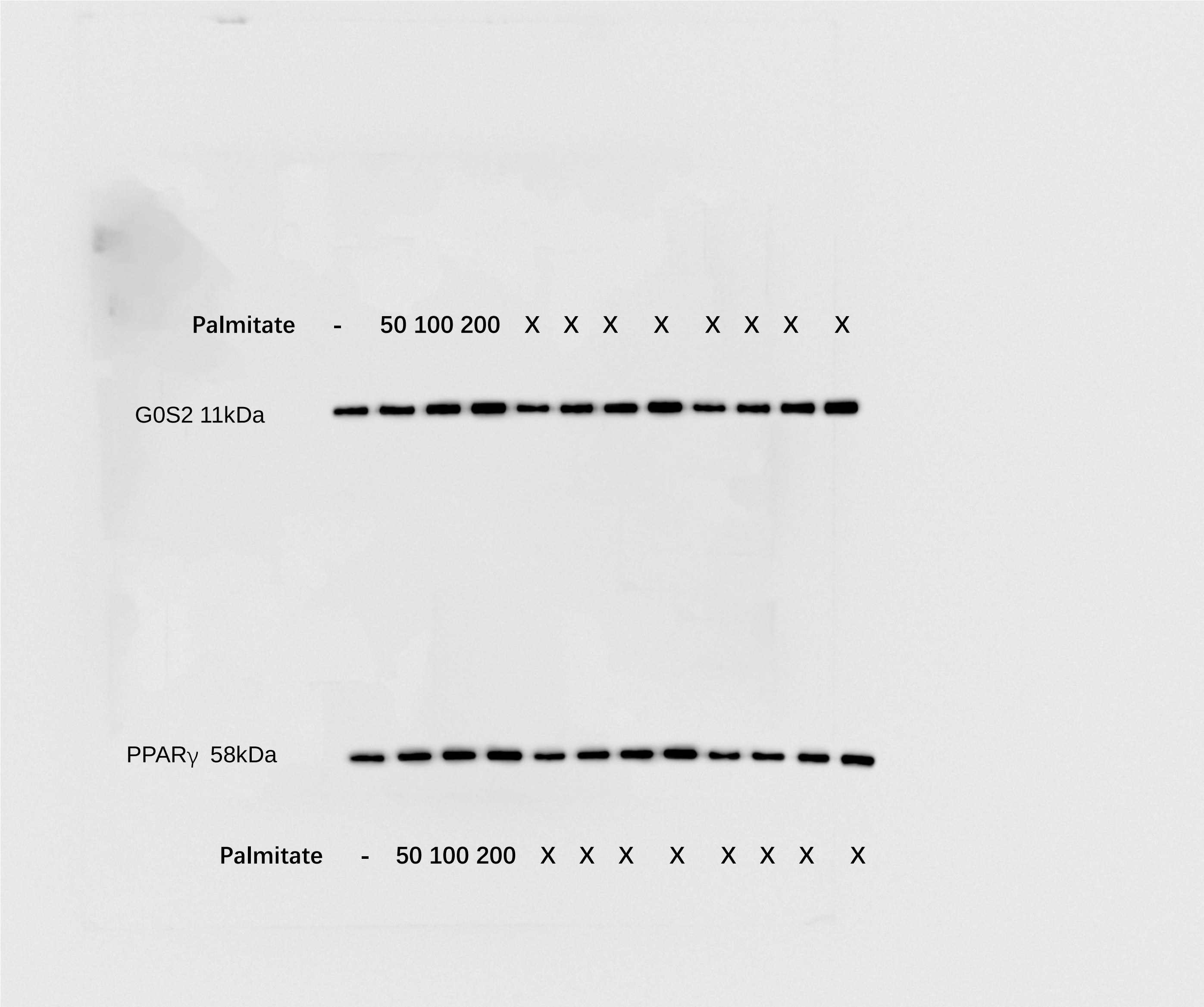
Palmitate - 50 100 200 X X X X X X X X
G0S2 11kDa
PPARγ 58kDa
Palmitate - 50 100 200 X X X X X X X X

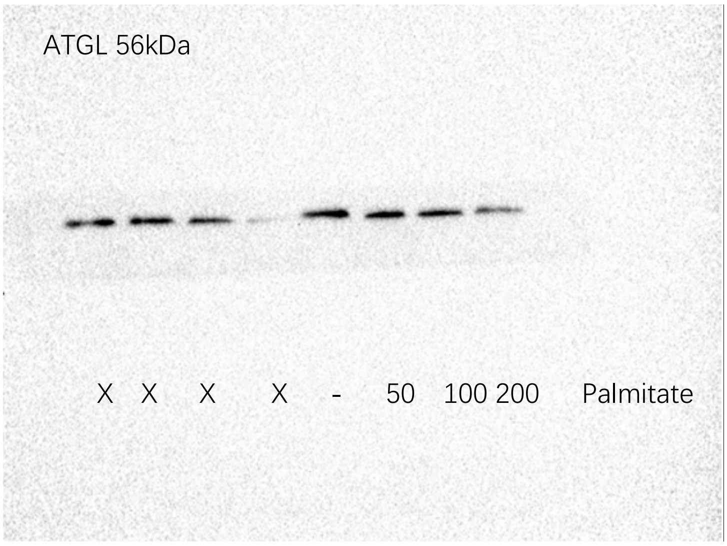
ATGL 56kDa
X X X X - 50 100 200 Palmitate

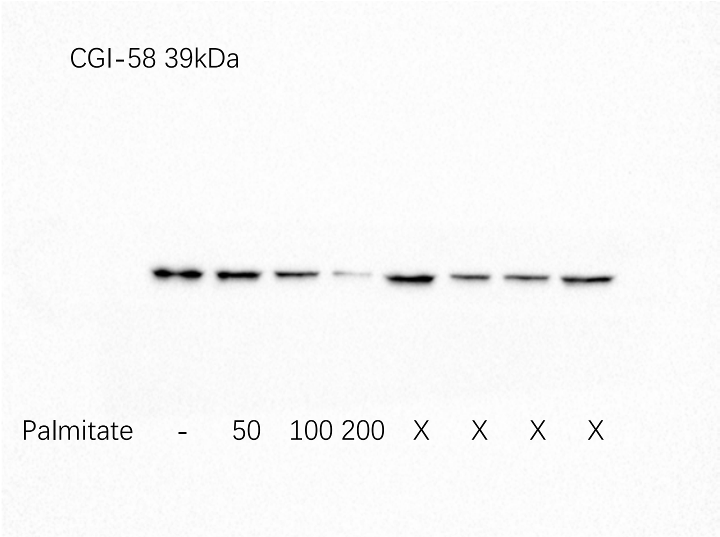
CGI-58 39kDa
Palmitate - 50 100 200 X X X X

Figure 3 A

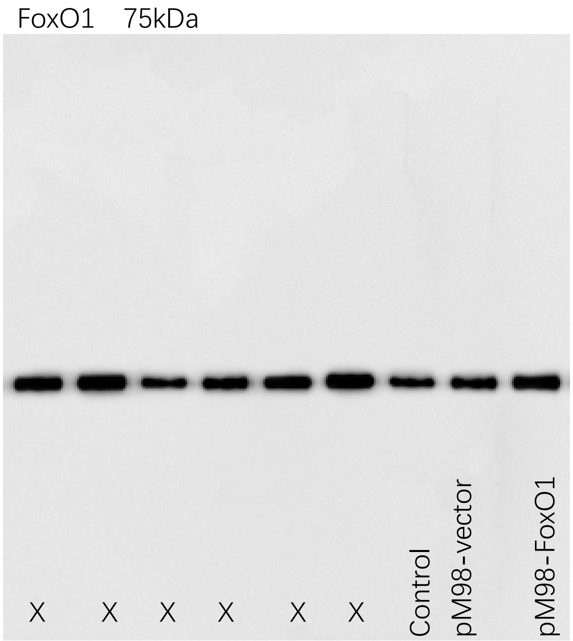
FoxO1 75kDa
X X X X X X Control pM98-vector pM98-FoxO1

β-actin 42kDa

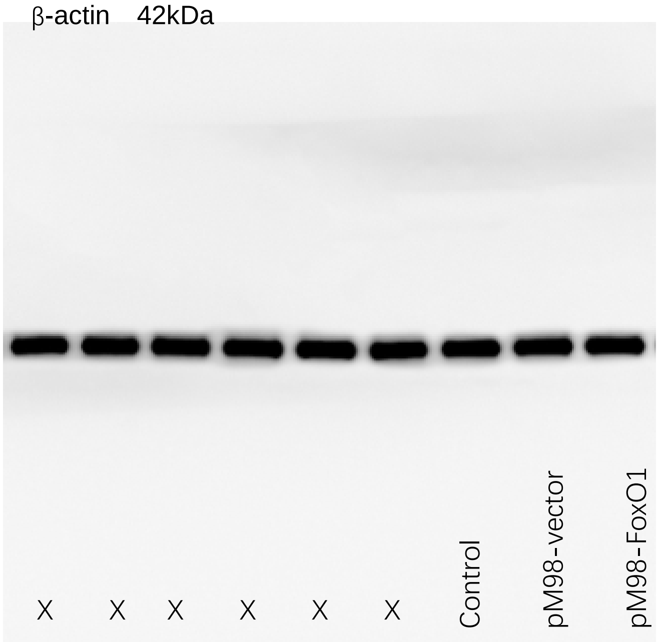

Figure 3 C

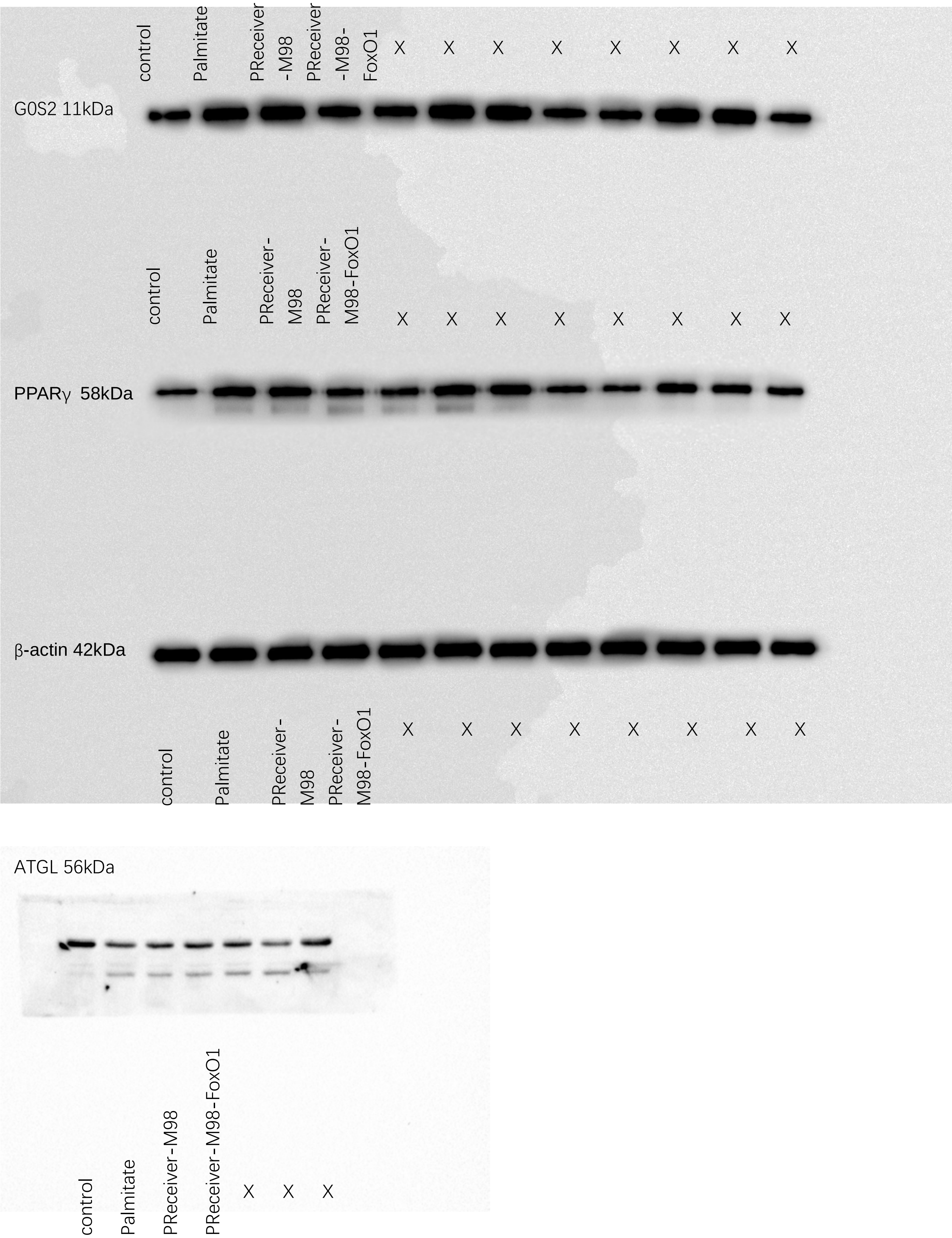
control
Palmitate
PReceiver
-M98
PReceiver
-M98-
FoxO1
X X X X X X X X
G0S2 11kDa
control
Palmitate
PReceiver-
M98
PReceiver-
M98-FoxO1
X X X X X X X X
PPARγ 58kDa
β-actin 42kDa
X X X X X X X X
control
Palmitate
PReceiver-
M98
PReceiver-
M98-FoxO1
ATGL 56kDa
control
Palmitate
PReceiver-M98
PReceiver-M98-FoxO1
X X X

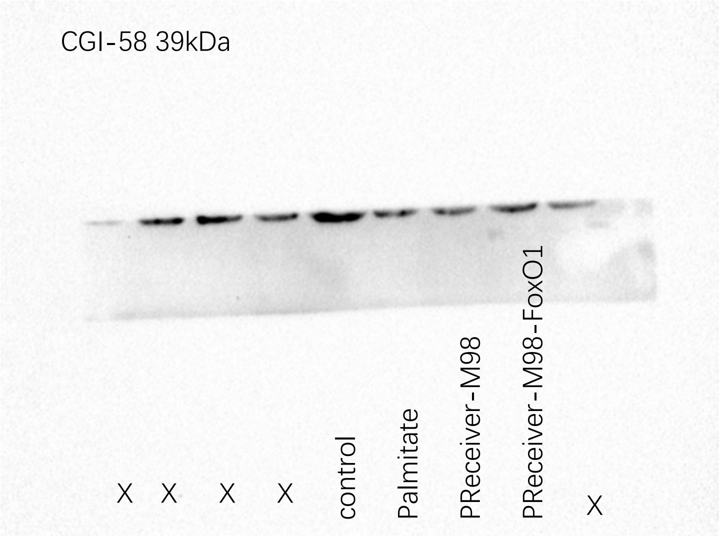

Figure 4

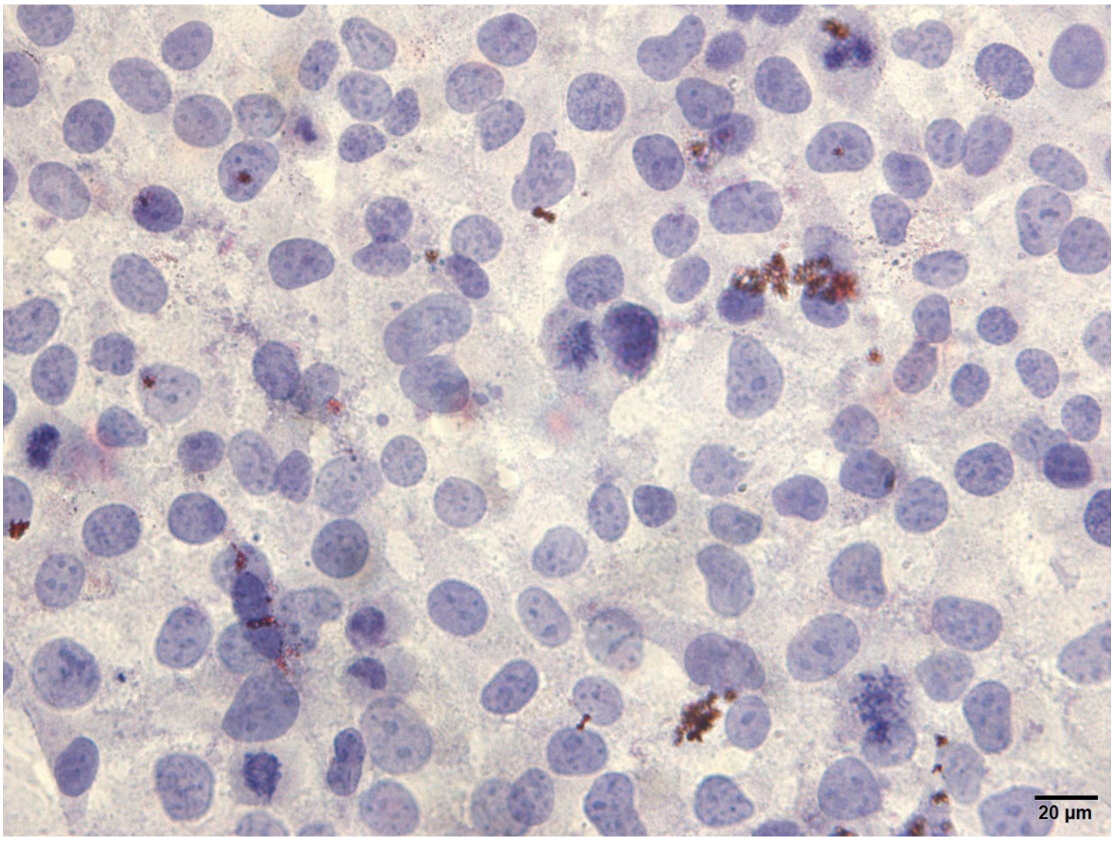

control

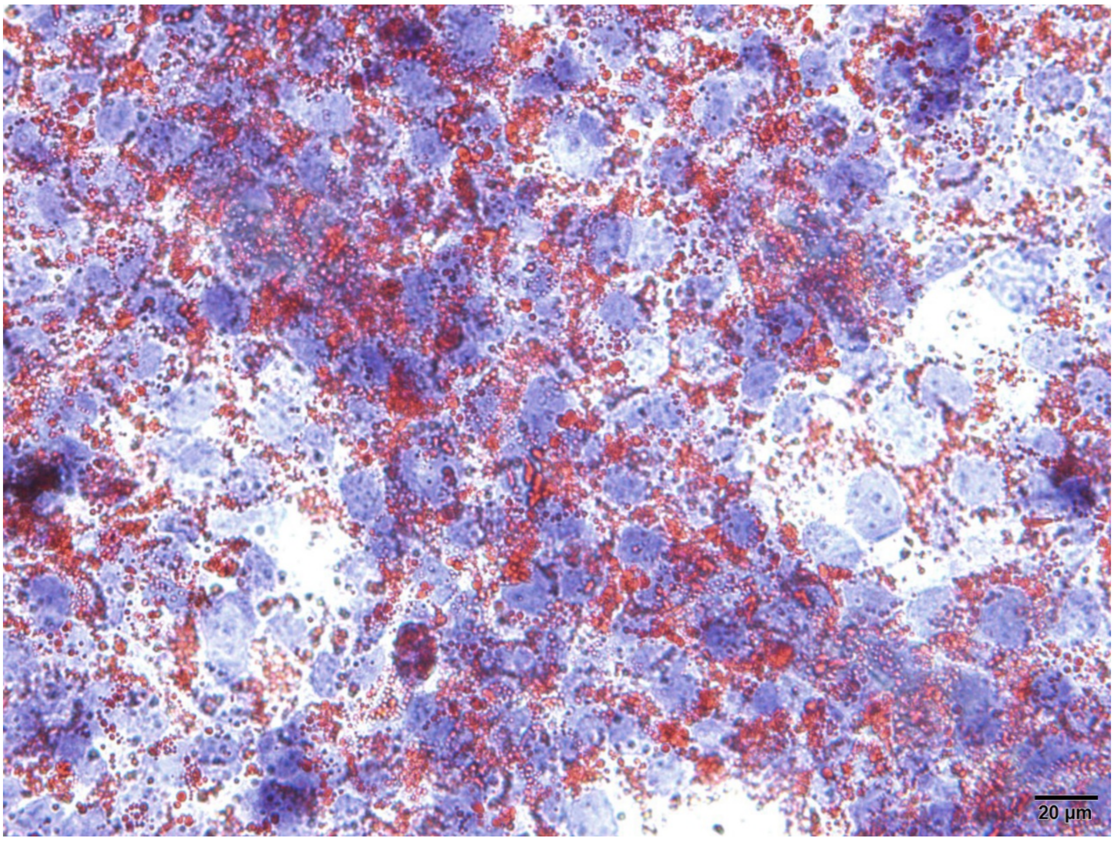

200µM palmitate

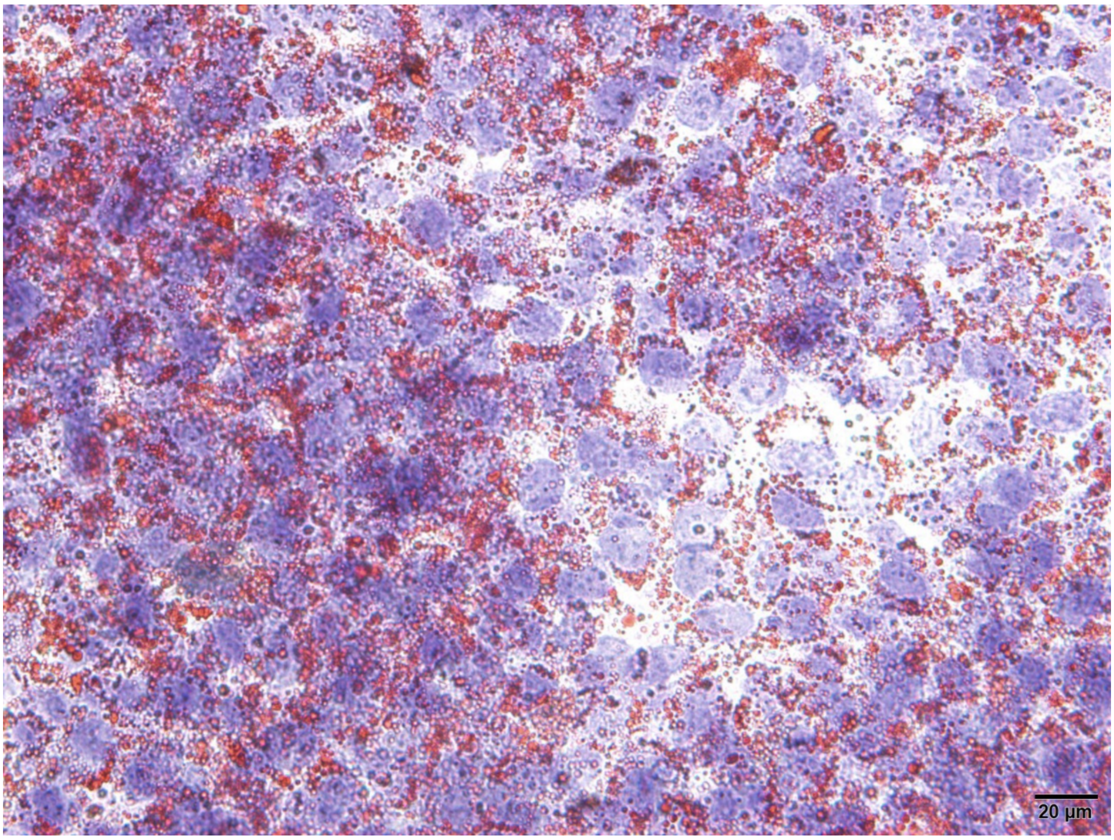

200µM palmitate+pReceiver-M98

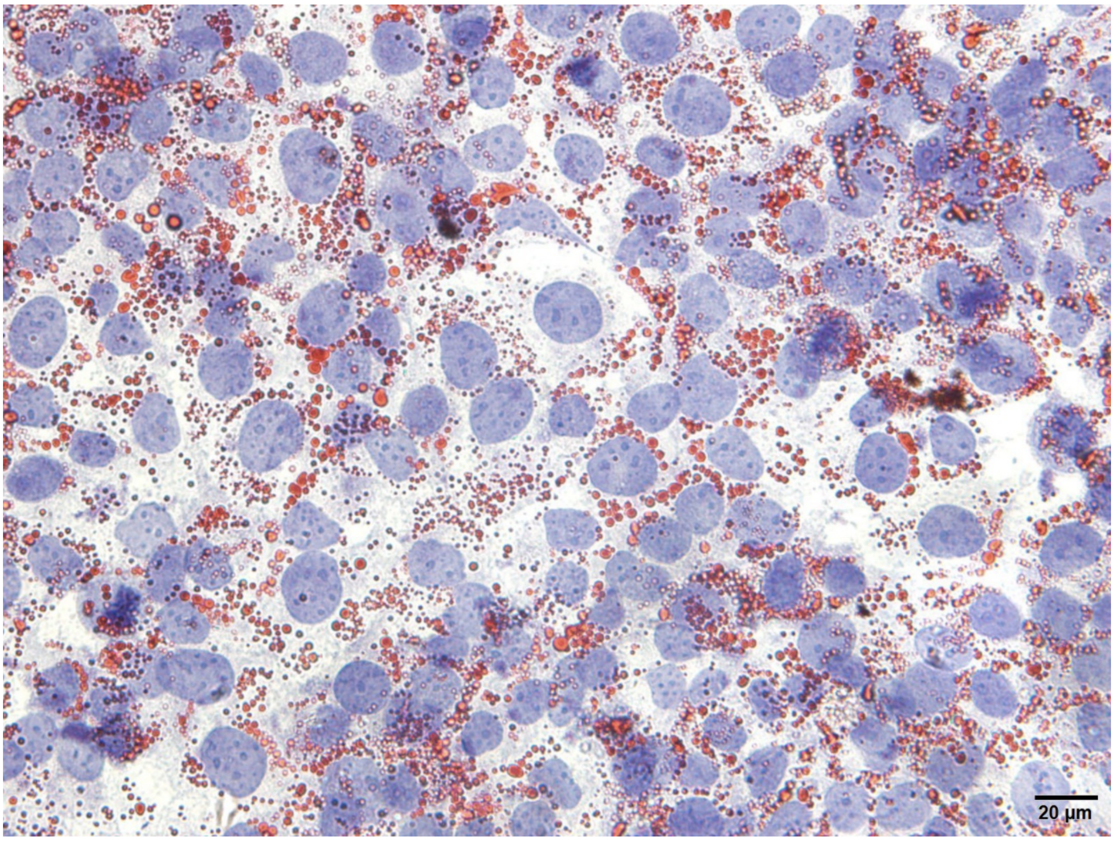

200µM palmitate+pReceiver-M98-FoxO1
